# Supplementary material for: Naïve individuals promote collective exploration in homing pigeons
Source: eLife. 2021 Dec 20;10:e68653. doi: 10.7554/eLife.68653 (PMC8687659; doi:10.7554/eLife.68653)
Supplement: Transparent reporting form [file elife-68653-transrepform1.docx]

***eLife’s* transparent reporting form**

We encourage authors to provide detailed information *within their submission* to facilitate the interpretation and replication of experiments. Authors can upload supporting documentation to indicate the use of appropriate reporting guidelines for health-related research (see [EQUATOR Network](about:blank)), life science research (see the BioSharing Information Resource), or the [ARRIVE guidelines](about:blank) for reporting work involving animal research. Where applicable, authors should refer to any relevant reporting standards documents in this form.

If you have any questions, please consult our Journal Policies and/or contact us: [editorial@elifesciences.org](about:blank).

**Sample-size estimation**

- You should state whether an appropriate sample size was computed when the study was being designed
- You should state the statistical method of sample size computation and any required assumptions
- If no explicit power analysis was used, you should describe how you decided what sample (replicate) size (number) to use

Please outline where this information can be found within the submission (e.g., sections or figure legends), or explain why this information doesn’t apply to your submission:

As (a large amount of) data were already available, no explicit power analysis was used but rather all available data have been used in the analysis. Significance of the results has been decided by means of statistical tests comparing original data with surrogate data obtained through a randomization procedure. See *Materials and Methods* for a detailed description of this procedure.

**Replicates**

- You should report how often each experiment was performed
- You should include a definition of biological versus technical replication
- The data obtained should be provided and sufficient information should be provided to indicate the number of independent biological and/or technical replicates
- If you encountered any outliers, you should describe how these were handled
- Criteria for exclusion/inclusion of data should be clearly stated
- High-throughput sequence data should be uploaded before submission, with a private link for reviewers provided (these are available from both GEO and ArrayExpress)

Please outline where this information can be found within the submission (e.g., sections or figure legends), or explain why this information doesn’t apply to your submission:

Detailed information about the experimental procedure, number of biological replicas as well as surrogate replicas can be found in the *Materials and Methods* section.

Raw and processed data as well as all source code used in the analysis has been deposited on *figshare* and it is privately availably to editors and reviewers (see data availability statement). Data will be made publicly available upon acceptance of the manuscript.

**Statistical reporting**

- Statistical analysis methods should be described and justified
- Raw data should be presented in figures whenever informative to do so (typically when N per group is less than 10)
- For each experiment, you should identify the statistical tests used, exact values of N, definitions of center, methods of multiple test correction, and dispersion and precision measures (e.g., mean, median, SD, SEM, confidence intervals; and, for the major substantive results, a measure of effect size (e.g., Pearson's r, Cohen's d)
- Report exact p-values wherever possible alongside the summary statistics and 95% confidence intervals. These should be reported for all key questions and not only when the p-value is less than 0.05.

Please outline where this information can be found within the submission (e.g., sections or figure legends), or explain why this information doesn’t apply to your submission:

Statistical analysis methods are described in sections *Results* and *Materials and Methods.* Detailed statistics are provided in the *Results* section as well as in the Supplementary material.

Raw data are reported only in some figures due to large sample size whenever informative and overlapped with aggregate statistics.

(For large datasets, or papers with a very large number of statistical tests, you may upload a single table file with tests, Ns, etc., with reference to sections in the manuscript.)

**Group allocation**

- Indicate how samples were allocated into experimental groups (in the case of clinical studies, please specify allocation to treatment method); if randomization was used, please also state if restricted randomization was applied
- Indicate if masking was used during group allocation, data collection and/or data analysis

Please outline where this information can be found within the submission (e.g., sections or figure legends), or explain why this information doesn’t apply to your submission:

Details of group composition can be found in *Materials and Methods: Experimental subjects and procedure*. A randomization procedure used to create surrogate datasets functioning as controls is described in *Materials and Methods: Measuring information transfer*.

No masking was necessary for experiments and/or data analysis.

**Additional data files (“source data”)**

- We encourage you to upload relevant additional data files, such as numerical data that are represented as a graph in a figure, or as a summary table
- Where provided, these should be in the most useful format, and they can be uploaded as “Source data” files linked to a main figure or table
- Include model definition files including the full list of parameters used
- Include code used for data analysis (e.g., R, MatLab)
- Avoid stating that data files are “available upon request”

Please indicate the figures or tables for which source data files have been provided:

Data and source code are provided for all figures and tables on *figshare*.

The *figshare* repository contains 1) raw data, 2) all intermediary steps of pre-processed data, and 3) R source code necessary to compute all intermediate and aggregated results of the analysis on the basis of raw data.
